# SaO_2_-SvO_2 _difference for risk stratification of patients with sepsis and septic shock

**DOI:** 10.1186/cc12955

**Published:** 2013-11-05

**Authors:** Fábio Ferreira Amorim, Adriell Ramalho Santana, Jaqueline Lima de Souza, Felipe Bozi Soares, Bárbara Magalhães Menezes, Mariana Pinheiro Barbosa de Araújo, Fernanda Vilas Bôas Araújo, Louise Cristhine de Carvalho Santos, Pedro Henrique Gomes Rocha, Alessandra Vasconcelos da Silva Paiva, Gabriel Kanhouche, Pedro Nery Ferreira Júnior, Alethea Patrícia Pontes Amorim, José Aires de Araújo Neto, Edmilson Bastos de Moura, Marcelo de Oliveira Maia

**Affiliations:** 1 Escola Superior de Ciências da Saúde, Brasília, Brazil; 2 Liga Acadêmica de Medicina Intensiva de Brasília, Brazil; 3 Hospital Santa Luzia, Brasília, Brazil

## Background

Assessment and monitoring of hemodynamics is a cornerstone in critically ill patients as hemodynamic alteration may become life-threatening in a few minutes [1,2]. This study aimed to determine whether the SaO_2_-SvO_2 _difference could be used as risk stratification for patients with sepsis and septic shock.

## Materials and methods

A retrospective cohort study conducted in the ICU of Hospital Santa Luzia, Brasilia, DF, Brazil, during 6 months. An arterial blood sample was collected at admission. Patients with sepsis were divided in two groups: survivors group (SG) and nonsurvivors group (NSG). The accuracy of SaO_2_-SvO_2 _difference to predict ICU mortality was measured with the area under the receiver operating characteristic curve.

## Results

A total of 131 patients with sepsis were enrolled, 11.5% (*n *= 15) with septic shock. Age was 66 ± 21 years, SAPS3: 37 ± 17, APACHE II: 14 ± 8, PaO_2_/FiO_2_: 342 ± 142 and SaO_2_/FiO_2_: 347 ± 109. ICU mortality was 18% (*n *= 23). The main sites of infections were respiratory (56.5%, *n *= 74), urinary (19%, *n *= 25) and cutaneous (7.6%, *n *= 10). Nonsurvivor patients had higher SaO_2_-SvO_2 _difference (26 ± 9 vs. 19 ± 9, *P *= 0.03). In the group of patients with septic shock, SaO_2_-SvO_2 _difference was also higher in nonsurvivors (29 ± 3 vs. 10 ± 2, *P *= 0.02). All patients with septic shock who died had SaO_2_-SvO_2 _difference greater than 25%. The SaO_2_-SvO_2 _difference area under ROC curve was 0.714 (95% CI 0.534 to 0.894).

## Conclusions

A higher SaO_2_-SvO_2 _difference is associated with mortality in patients with sepsis, especially in patients with septic shock.
